# Multiscale Carbon Fibre–Carbon Nanotube Composites of Poly(Vinyl Chloride)—An Evaluation of Their Properties and Structure

**DOI:** 10.3390/molecules29071479

**Published:** 2024-03-26

**Authors:** Katarzyna Skórczewska, Sławomir Wilczewski, Krzysztof Lewandowski

**Affiliations:** Faculty of Chemical Technology and Engineering, Bydgoszcz University of Science and Technology, Seminaryjna 3, Street, 85-326 Bydgoszcz, Poland; slawomir.wilczewski@pbs.edu.pl (S.W.); krzysztof.lewandowski@pbs.edu.pl (K.L.)

**Keywords:** poly(vinyl chloride (PVC), hybrid filler system, multiscale composite, carbon fibres (CFs), carbon nanotube (CNT), hybrid structure, properties

## Abstract

To date, there has been limited information in the literature on the application of carbon fibre-carbon nanotube systems for the modification of poly(vinyl chloride) (PVC) matrixes by micro- and nanometric fillers and an evaluation of the properties of the unique materials produced. This paper presents the results of newly designed unique multiscale composites. The advantages of the simultaneous use of carbon fibres (CFs) and carbon nanotubes (CNTs) in PVC modification are discussed. To increase the dispersibility of the nanofiller, CFs together with nanotubes were subjected to a sonication process. The resulting material was introduced into PVC blends, which were processed by extrusion. The ratio of components in the hybrid filler with CF_CNT was 20:1, and its proportion in the PVC matrix was 1, 5, and 10 wt.%, respectively. Comparatively, PVC composites modified only with carbon fibres were obtained. The structure, thermal, electrical, and mechanical properties and swelling resistance of the composites were studied. The study showed a favourable homogeneous dispersion of nanotubes in the PVC matrix. This enabled effective modification of the structure at the nanometric level and the formation of an interpenetrating network of well-dispersed hybrid filler, as evidenced by a decrease in volume resistivity and improvement in swelling resistance, as well as an increase in glass transition temperature in the case of PVC/CF_CNT composites.

## 1. Introduction

Standard carbon fibre-reinforced polymer composites (CFRPs) are widely used materials, often displacing metals and their alloys due to their low density, corrosion resistance, high mechanical properties such as stiffness and strength, and availability and relative ease of formability [1]. Ensuring such high mechanical properties depends on several factors, and the main one is the ability to transfer loads at the interface, and thus, allow interfacial interactions. Polyester and epoxy resins, as well as thermoplastics such as polyamides (PAs), polycarbonates (PCs), or acrylonitrile–butadiene–styrene (ABS), are most commonly used as matrices [2]. On the other hand, there is little information on the use of PVC as a matrix for such composites [3,4], which is due to the difficult processing of this polymer.

Multiscale polymer composites containing simultaneously more than one discontinuous phase in the form of a microfiller and a nanofiller dispersed in a continuous polymer matrix are of increasing interest [5,6,7,8,9,10,11]. Of particular interest in terms of novel properties are composites where the micro- and nanofillers are carbon structures [12,13]. One such combination may be the simultaneous use of graphene or carbon nanotubes together with carbon fibres [9,14,15,16]. Such a system can lead to simultaneous micro- and nanometric modification and, thus, to the use of the properties of micro- and nanostructures in a one-polymer composite, for example, resulting in a material with significantly improved mechanical properties and at the same time increased chemical or thermal resistance and new properties unattainable with a conventional filler. The synergistic effect of the fillers can trigger additional modification mechanisms not available with conventional methods, resulting in a material with extended functionalities. Such hybrid composites can achieve much more favourable properties with a reduced proportion of microfiller and a small proportion of nanofiller.

The effectiveness of property changes in multiscale composites is challenging due to the appropriate dispersion of the two types of fillers, their interaction with each other, and interactions with the polymer matrix. In addition, the morphology of the nanofiller (high aspect ratio and tendency to agglomerate) or microfiller (lack of suitable surface functional groups, lack of roughness and compatibility) and its properties may result in smaller changes than expected. Finding the relationship between the specific hybrid structure produced in such composites and the properties is often an object of research [2,14,17].

Carbon nanotubes as rolled-up hexagonal graphene sheets are interesting modifiers for the production of modern polymeric composite materials [18]. The use of CNTs in this direction is supported by the remarkable properties of these nanostructures, such as high mechanical strength and elasticity, high chemical and thermal stability, surprising electrical properties, and thermal conductivity [19]. The use of carbon nanotubes for the modification of carbon fibre composites is aimed at improving interfacial interactions [20,21], increasing the specific surface area of the CFs, and thus improving the load transfer capacity of the matrix filler. This strategy makes it possible to obtain hybrid composites with new properties that depend on the micro- and nanometric structures obtained.

Poly(vinyl chloride) is a polymer widely used in many industries. It is characterised by good resistance to water and atmospheric conditions, and thus, its properties remain unchanged over long periods of use. The polymer can be easily modified in terms of its functional properties. It is used in the manufacture of long-life components such as window profiles, cable insulation, drainage pipes, and decking, as well as packaging material, e.g., for pharmaceuticals or blood bags [22]. The polymer also serves as a matrix for modern polymer nanocomposites modified with graphene [23,24,25], nanocarbon [12], and other nanoparticles [26]. Fibres of natural origin are also used to modify PVC [27], but there is little information on the use of carbon fibres [3,28] or glass fibres [29] in this field.

The current literature reveals little information on PVC composites with CFs and CNTs combined [30,31]. In our previous work [30], we used poly(vinyl acetate) PVAc as sizing to improve CF-PVC interfacial interactions. The results presented here show the use of CFs and CNTs as fillers to obtain micro- and nano-reinforced composites. In addition, our previous work shows that obtaining a homogeneous dispersion of CNTs in PVC is very difficult and requires multistep fabrication methods. Therefore, in the results presented here, CFs served, as it were, as an additional factor dispersing CNTs and reducing their aggregates, which could be translated into the quality of the final dispersion of the nanofiller in the matrix.

## 2. Results and Discussion

### 2.1. Morphology and Structural Characterization

**Figure 1:**
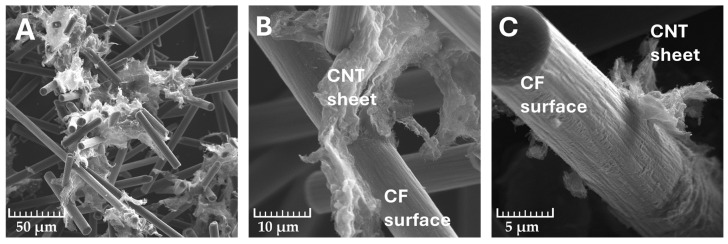
SEM images of carbon filler system CF/CNT ((**A**)—mag. 1000×; (**B**)—mag. 5000×; (**C**)—mag. 10,000×).

The SEM images (Figure 1) show the morphology of the filler obtained after mixing them according to the described methodology at 1000×, 5000×, and 50,000× magnification. The resulting system of CF microfiller and CNT nanofiller is made up of CF fibres with carbon nanotubes sandwiched between them in the form of sheets of paper-like layers, some of which wrap around the surface of the carbon fibres. However, the surface structure of the fibre is mostly unchanged.

The aggregates of the applied CNTs described in the literature resemble a “bird’s nest” of approximately 10 μm to 1 mm in size with a strong tight bundle flush [32]. The joint mixing of CFs and CNTs, however, increased the surface area of the nanofiller in the form of the formation of its loose sheets with a looser, more jagged, and accessible structure, and thus may translate into a favourable dispersion in the PVC matrix, allowing the formation of an interpenetrating network of micro- and nanometric filler systems.

**Figure 2:**
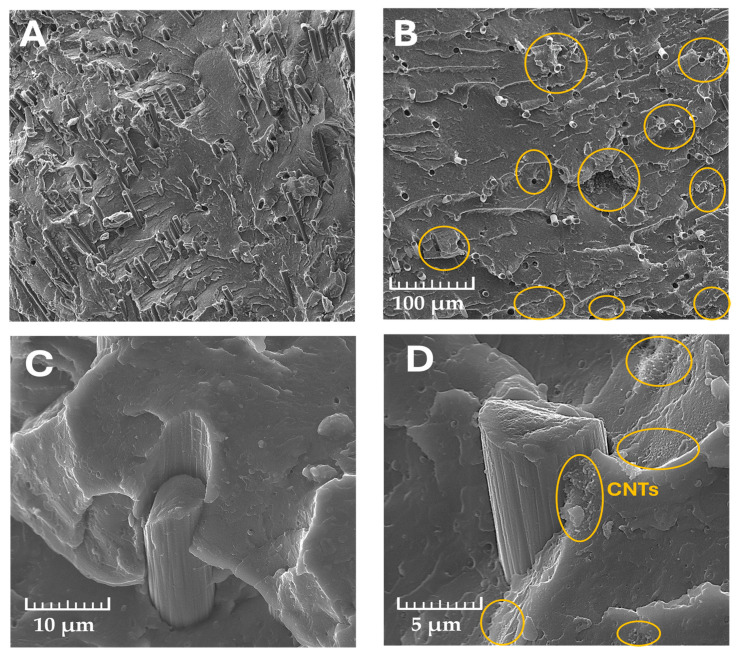
SEM observations of cryogenic fracture of PVC/5CF (**A**) and PVC/5CF_CNT (**B**) composites and the fibre–matrix interface in PVC/CF; (**C**) fibre–matrix interface in PVC/CF_CNT (**A**,**B**,**D**)—mag. 500×; (**C**)—mag. 5000×; (**D**)—mag. 10,000×. Yellow circles indicate CNT placement areas.

Figure 2 shows the cryogenic cross-section of the PVC/5CF and PVC/5CF_CNT composites at 500× magnification and a magnified image of the fibre–matrix interface in the matrix–CF system and in the matrix–CF–CNT system (Figure 2C,D).

The distribution of fibres in the composite is mostly homogeneous and their arrangement in the matrix shows no orientation. It can be observed that in the case of the PVC/CF composite, voids of fibres located horizontally to the breakthrough surface are visible, which indicates limited adhesion at the phase boundary in this system. On the surface of the cryogenic PVC/CF_CNT breakthrough, the mode of fracture is slightly different; namely, the majority of the destruction occurred at the diameter of the fibre and not at the point of its bonding to the polymer matrix. However, even here, areas of pulling the fibre out of the matrix are also visible, indicating insufficient bonding to the polymer matrix. This surface also highlights the presence of CNT aggregates (marked with a yellow circle) distributed between the fibres. Such a placement is likely to enhance their contact potential and thus shape the electron transfer capacity of the material.

The method of joining the filler to the matrix can be correlated with the mechanical properties of the composites. A lack of sufficient adhesion at the interface results in inefficient load transfer between the matrix and filler and thus can be linked to a reduction in tensile strength. Caverns are present at the interface between the fibre and the polymer matrix, which is more pronounced in composites containing only CFs. When a nanofiller is additionally used, areas of PVC-CNTs with increased adhesion to the carbon fibre surface can be observed, which anchor the fibre to the PVC matrix, thus improving its adhesion to the PVC matrix. Similar observations of the PVC/CF_CNT structure were described in our work [30], where the deposition of CNTs on the CF surface with PVAc helped to increase the adhesion of the fibre to the PVC matrix.

### 2.2. Analysis of Glass Transition Temperature by Differential Scanning Calorimetry (DSC) and Dynamical Mechanical Analysis (DMA) Methods

**Figure 3:**
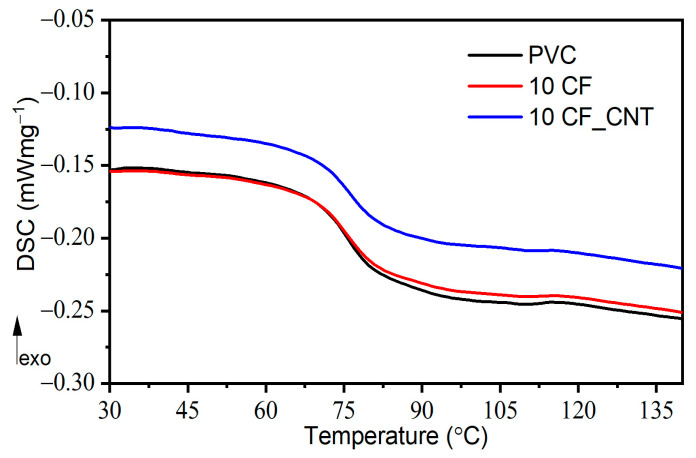
The glass transition region of PVC and PVC composites contains 10 wt.% of CFs and CF_CNT investigated by DSC.

**Figure 4:**
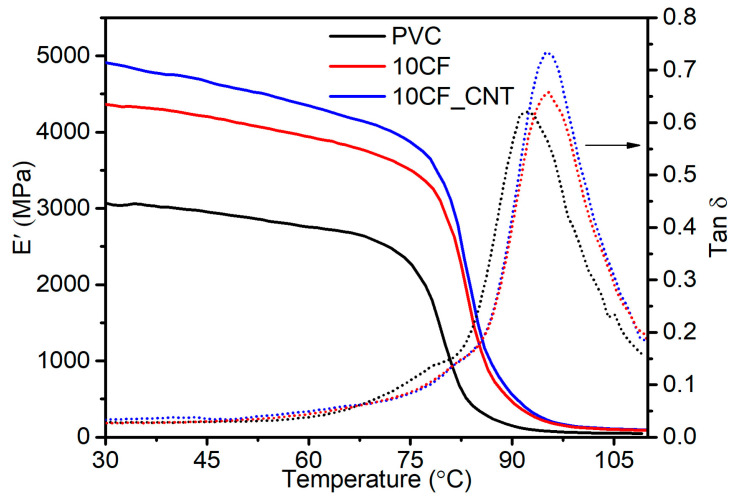
Example DMA thermograms PVC and PVC composites contain 10 wt.% of CFs and CF_CNT, *E*’ (straight line) tan *δ* (dotted line).

Carbon nanotubes, due to their nanometric size and homogeneous dispersion in the matrix, have a more effective influence on intermolecular interactions compared to microfillers, which can be manifested by blocking the molecular movements of macromolecule segments during the glass transition (*T_g_*) even at low nanofiller content in the PVC matrix. The increase in *T_g_* values can therefore be linked to an increase in physical interactions between the carbon nanotubes and the matrix and the presence of a new phase (boundary layer) between the polymer macromolecules and the nanotube surface [33]. Figure 3 shows examples of DSC thermograms in the range of the glass transition temperature analysed. Example DMA thermograms are shown in Figure 4. A summary of the results of the glass transition temperature analysis determined by DSC and DMA is presented in Table 1.

The results of DSC analysis of *T_g_* for PVC/CF composites show that there is no clear change in glass transition temperature with increasing CF content, and its value remains similar to that of the unmodified matrix, i.e., about 75.7 °C.

In assessing *T_g_* changes by DMA, a slight increase in *T_g_* accompanied by a rapid fall in modulus and a maximum tan *δ* value was found with an increase in CF content. The difference between the DSC and DMA test results may be due to the different effects measured during the test. DSC measurement takes into account only a small piece of the measurement sample, whereas with DMA, we obtain results from a much larger sample. When relatively large filler particles are used, compared to the polymer macromolecules, several macroscopic interactions that are outside the measuring range of DSC are revealed.

From the point of view of practical applications, the use of the DMA method for the assessment of the glass transition temperature, which determines the change in mechanical properties during the glass transition, seems to be more relevant in the evaluation of composites than the thermal properties using the DSC method. Therefore, based on the results of the DMA method indicating an increase in the *T_g_* value, it can be concluded that the proposed method of PVC modification by applying the CF_CNT filler system favourably influences the increase in the range of application temperature of the produced composites in comparison with unmodified PVC.

Also, for PA composites with CFs, no change in glass transition temperature was observed with increasing CF content and their length [34]. For PP/CF composites containing 10 wt.% of CFs with a length of 10 mm, a shift in the maximum tan δ value indicating an increase in *T_g_* to a higher temperature was noted, while for other sizes of CFs, no considerable change was observed [35]. Other reports on PBT/30%CF modifications showed a 2 °C increase in *T_g_* values. This was caused by a slight reduction in segmentation movements [36].

Comparing the *T_g_* values of the prepared PVC/CF and PVC/CF_CNT composites, it can be concluded that the addition of the nanomodifier slightly shifts the *T_g_* towards higher values. This is a result of additional interactions at the nanofiller–macromolecule interface, which reduces the mobility of the polymer chain segments and consequently increases the *T_g_* values. Moreover, the presence of dispersed nanofillers between the PVC macromolecules reduces the availability of intermolecular free space (through an increase in packing density), which would allow the rotation of the macromolecule segment at a lower temperature [37], and therefore, more energy (higher temperature) is needed for the segmentation movement to occur and, hence, an increase in *T_g_* values.

Importantly, the method of producing hybrid PVC/GF_CNT composites has a significant impact on their *T_g_* value. The use of additional coupling agents compatible with PVC may reduce its value. Slightly lower *T_g_* values when using the hybrid filler CF/CNT with PVAc as a sizing agent were described in our earlier work [30]. PVAc (*T_g_* value in the range 30–45 °C) and PVC show some compatibility, as a consequence of which an averaged glass transition temperature shift towards lower values may occur [38]. Nevertheless, a slight increase in *T_g_* values caused by the influence of CNTs on PVC macromolecules was also observed.

### 2.3. Results of Dynamical Mechanical Analysis

Table 2 summarises the values of the *E’* modulus determined at different temperature values and the coefficient C [39], calculated according to Equation (1), which reflects the fibre reinforcement efficiency.

Regardless of the filler used, its introduction into the polymer increased the modulus values of the composites in the glassy state as well as in the highly elastic state above 90 °C. These values were higher for PVC/CF_CNT composites. As the temperature increased, the modulus value of the composites decreased, but to a slightly lower level for the PVC/CF_CNT composites. The value of *E’* modulus increased with increasing filler quantity in the matrix. Thus, compared to unmodified PVC, the *E′* value at 30 °C (*E′* 30 °C) increased by 42% and 60% for the PVC/10CF and PVC/10CF_CNT composites, respectively.

The *E′* 90 °C values of PVC/10CF and PVC/10CF_CNT compared to the unmodified matrix increased by 211% and 275%, respectively. Such high increases in *E′* 90 °C values can be associated with more favourable elastic behaviour in the viscoelastic region [40].

The effectiveness of fillers on the moduli *E*’ of the composites can be represented by a coefficient C [39]. The higher the value of the coefficient C, the lower the effect of the filler on the increase in the retaining modulus. As the filler content in the matrix increases, the value of the coefficient C decreases. Lower values were recorded for PVC/CF_CNT composites compared to composites with the same content of CFs only.

The modulus *E’* indicates the elastic energy stored in the material and is strongly dependent on its composition, morphology, and geometrical properties. One important factor influencing the value of the modulus in the glassy state is the intermolecular forces of the matrix and the way the macromolecules are arranged in the polymer. In the case of composites, the proportion of filler and the interfacial adhesion that allows the transfer of stresses from the polymer matrix to the reinforcement is also important.

Based on the obtained values of changes in modulus and coefficient C reflecting the fibre reinforcement efficiency, it can be concluded that the micro- and nanofiller system is more effective in increasing the intermolecular interactions, which can be associated with the presence of well-dispersed carbon nanotubes. The presence of well-dispersed CNTs in the PVC matrix results in an increase in intermolecular interactions through the possibility of macromolecule stiffening. This may also confirm the observations and conclusions describing an increase in the glass transition temperature values of PVC/CF_CNT composites. On the other hand, in the case of epoxy composites containing 0.3% CNTs 1% CFs, even though they reported a 41% increase in the storage modulus with no change in the tan *δ* peak, Rahmanian et al. [41] indicated that there was no effect of the CNT and CF filler system on the mobility of the polymer chains.

### 2.4. Results of Mechanical Properties

**Figure 5:**
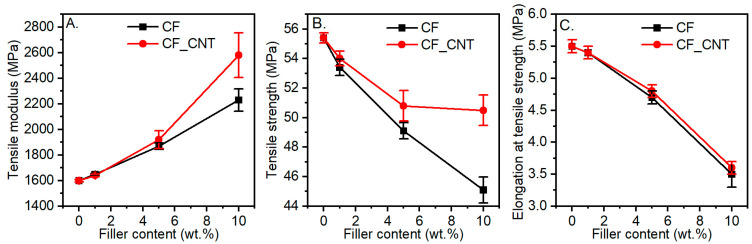
Mechanical properties of the PVC/CF and PVC/CF_CNT composites as a function of filler concentration. (**A**) Tensile modulus; (**B**) tensile strength; (**C**) elongation at tensile strength.

Figure 5 presents the results of testing the following tensile properties depending on the share of the hybrid filler in the matrix: the tensile modulus (*E_t_*), tensile strength (*σ_M_)*, and elongation at maximum load (*ε_M_*).

As the amount of filler in the matrix increased, the *E_t_* value increased. Composites with additional CNTs showed a more pronounced increase in modulus compared to those modified only with CFs. At the maximum filler content in the matrix, an increase in Et of 61% was observed in comparison with unmodified PVC, and this was 15% higher in comparison with PVC/10CF.

An increase in elastic modulus is indicative of the interactions occurring between the polymer matrix and the filler. Effective distribution of the filler through the polymer chains is key to constructing interconnected 3D structures of fillers. Such nanoscale reinforcements transfer loads between the polymer and filler more efficiently. In addition, the predominance of CNT-PVC interfacial areas (due to the nanometric dimensions of CNTs) and their overlap and penetration with CF-PVC interfacial areas provide more efficient load transfer between the matrix and the reinforcing material, thus enabling a better utilisation of the reinforcing effect of micrometric carbon fibres [42].

The observed reinforcement effect occurred only in a limited deformation range. The structure of the composite, which consisted largely of polymer, and the interfacial interactions were insufficient for complete stress transfer. Also, at higher deformations, the polymer–filler interactions were disrupted. This manifested itself as a reduction in the elongation values *ε_M_* and *σ_M_*. These parameters decreased independently of the filler type with the proportion in the matrix. The composite additionally containing CNT-modified carbon fibre was characterised by a lower decrease in strength, which should be considered a very favourable property. Compared to PVC, the composite containing 10 wt.% of CFs was characterised by a *σ_M_* value lower by approx. 18% and the PVC/CF_CNT composite by 8%. Compared to the PVC/10CF composite, the composite containing additional CNTs obtained a *σ_M_* value about 12% higher. Furthermore, the CF filler significantly reduced the orientation ability of the PVC macromolecules, which explains the decreases in *σ_M_* and *ε_M_*.

However, it is noticeable that composites containing additional CNTs in the matrix have higher property values compared to PVC composites containing only CFs [14]. L. Mészáros et al. [17] observed no significant changes in the tensile strength, tensile modulus, and elongation at break for polylactide PLA/CF composites. In contrast, they noted an increase in mechanical properties when the PLA matrix was simultaneously modified with CNTs and CFs, and this effect was related to the fact that the carbon nanotubes improved the load transfer between the matrix and the carbon fibres. The overlapping interphase regions of the nano- and micro-reinforcements resulted in improved load transfer between the carbon fibre and the matrix.

Compared to PVC, however, no significant improvement in mechanical properties was achieved for the PVC/CF_CNT composites. The likely reason could be the CNT aggregates observed in the SEM images, which reduce the expected strengthening effect. A completely homogeneous dispersion of CNTs in the polymer is often difficult to achieve and has been widely cited in the literature as a reason for the lack of improvement in composite properties [21].

### 2.5. Analysis of Thermal Stability by TGA and Time of Thermal Stability by Congo Red Test Method

**Figure 6:**
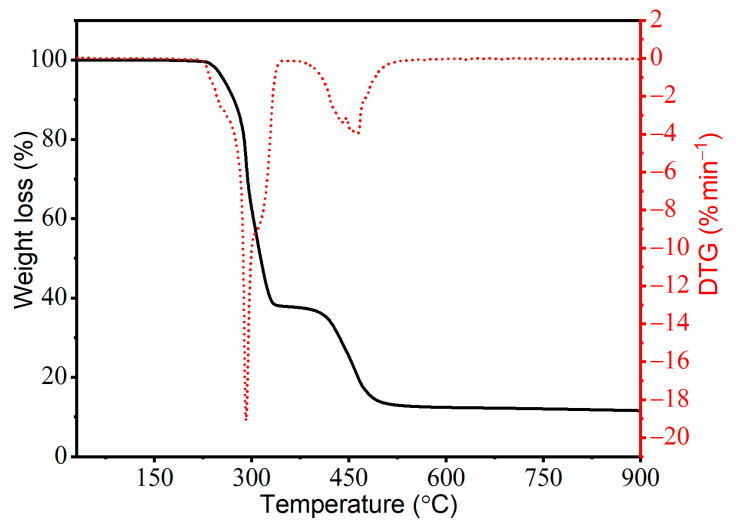
Thermograms of TG (black solid line) and DTG (red dotted line) of materials (PVC, PVC CF, and PVC CF/CNT) tested in a nitrogen atmosphere.

Figure 6 shows the TGA and derivative thermogravimetric (DTG) curve of the unmodified PVC. The decomposition of PVC and its composites occurs similarly, being a two-step process, independently of the analysed composition. The first stage of decomposition shows the dehydrochlorination of PVC macromolecules and release of gaseous hydrogen chloride (HCl) [43,44,45], while the next stage is a further decomposition of the resulting polymeric diene cross-linked structure [46,47], as a result of which the residual mass remains undecomposed.

Regardless of the type of filler and its quantity, the composites obtained were characterised by similar thermal stability; i.e., the characteristic readings have similar values compared to the unmodified PVC (Table 3). Comparing the *T*_5_ values, a slight increase can be observed with an increase in the amount of fillers in the matrix, which was slightly higher when only CFs were used. The maximum *T*_5_ value was characterised by the composite containing 10 wt.% of CFs (an increase of 7.9 °C in comparison to PVC). The *T*_10_ and *T*_50_ values of the composites were also higher than those of the unmodified matrix, with the same trend of change as for *T*_5_. On this basis, it can be concluded that the addition of CFs and CF_CNT has a slight effect on increasing the thermal stability of PVC composites, but the CF_CNT filler system improves this property to a minor extent. A significant increase in thermal stability was observed in the case of polypropylene (PP) composites containing both CF- and CF+CNT-modified maleic anhydride-grafted PP according to the authors due to the increase in a dense and continuous hybrid CF/CNT protective layer preventing PP decomposition [48].

The time of thermal stability assessed by the Congo red test [49,50] allows the determination of the stability time after which the PVC matrix decomposes with rapid HCl release, thus causing the discolouration of the indicator paper. The thermal stability time of the composites increased slightly with increasing filler content, irrespective of its type in the matrix. A maximum increase of about 2 min was observed for PVC composites with 10CF_CNT. These effects suggest a favourable effect of fillers on increasing the thermal stability time of macromolecules. This may be due to an increase in the heat uptake capacity of the dispersed nano- and microfillers throughout the volume, which may contribute to an increase in the thermal stability of the macromolecules by prolonging the initiation of the decomposition reaction of the PVC macromolecules.

### 2.6. Electrical Properties

CNTs have remarkable electrical properties and, with their high aspect ratio of length to diameter, are an ideal candidate for creating conductive materials. The use of carbon nanotubes in the PVC matrix is a well-known and effective way of modifying the electrical properties. The effects of modification of this property are already achieved with a small proportion of nanofiller in the matrix, provided that an appropriate spatial percolation network is obtained.

The modification of PVC by CFs also leads to changes in the electrical properties of the composites. However, this change is strictly dependent on the quality of the fibre, its length, and the significant proportion in the polymer matrix, which is incomparably higher than in the case of nanofillers.

The effectiveness of the change in electrical properties of composites at low carbon fibre content and the presence of CNTs has been reported in the literature [51]. Such a procedure makes it possible to produce an additional nanofiller system, which, by forming additional hybrid conduction networks with microfillers, intensifies the conductivity with a negligible proportion of fillers in the matrix [6,52,53].

**Figure 7:**
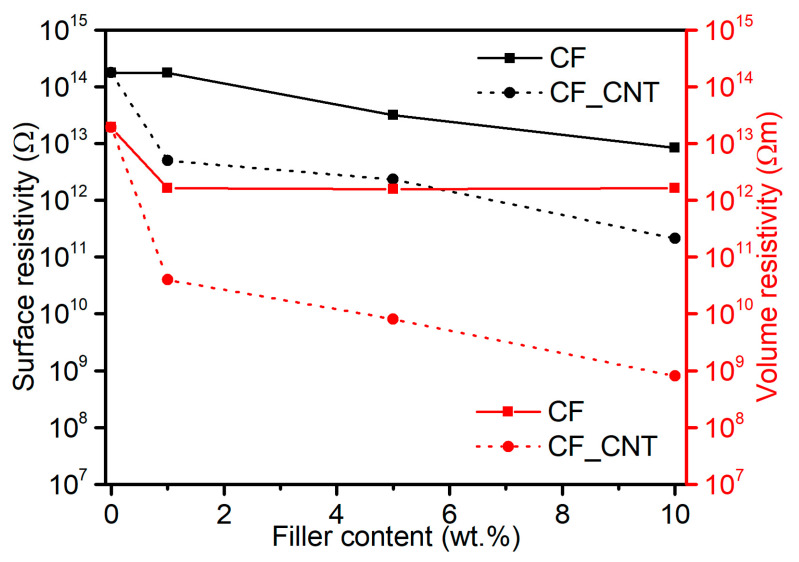
Volume (*R_v_*) and surface resistivity (*R_s_*) of PVC materials as a function of hybrid filler content.

The change in volume and surface resistivity of PVC/CF and PVC/CF_CNT composites is shown in Figure 7.

The surface resistivity (*R_s_*) value of unmodified PVC was 1.8 × 10^14^ Ω, and the introduction of CFs into the matrix resulted in a reduction in resistivity of Rs ranging from 1.7 × 10^14^ Ω to 8.3 × 10^12^ Ω for the composite containing 1% CFs and 10% CFs, respectively. Similar effects of a slight reduction in resistivity were reported for PVC/CF composites with a weight ratio of 1:1, respectively, produced by solution casting [3]. On the other hand, in the paper [54], it was indicated that electrical conductivity increased with increasing weight fraction of fillers and the type of carbon filler, which was used for the modification of PVC in the range of up to 50 wt.%. For PVC composites additionally containing CNTs, the *R_s_* value ranged from 5 × 10^12^ Ω to 2.1 × 10^11^ Ω. The reduction in resistivity *R_s_* was due to the presence of CNTs and the generation of additional conduction paths.

The volume resistivity (*R_v_*) value of unmodified PVC was 1.9 × 10^13^ Ωm; the introduction of CFs, irrespective of their proportion, resulted in a reduction of one order of magnitude to 1.2 × 10^12^ Ω. Higher resistivity changes were observed for the PVC/CF_CNT composites, especially those containing 10 wt.% CF_CNT, where reductions in resistivity of five orders of magnitude compared to PVC and four orders of magnitude compared to PVC/10CF were achieved. These results indicate the formation of a spatial structure of interpenetrating micro- and nanofillers capable of efficient electron transfer. It should be noted that in the case of the PVC/10CF_CNT composite, the proportion of nanofiller was 0.46 wt.%. These resistivity results classify the material as an anti-electrostatic material.

The CNT dispersion is visible during scanning electron microscopy analysis (SEM) in the form of thin layers/sheets; despite the lack of individual dispersion, it is characterised by homogeneity, forming a bridge for electron flow inside the composite, and together with CFs uniformly distributed in the PVC matrix, is the basis for the formation of conduction paths in the PVC matrix composite. The generated dual system of pathways consisting of connections between nanofillers, microfillers, and nano-microfillers is involved in electron conduction changing the properties of PVC classified as an insulating material to a composite classified as an anti-electrostatic material [55]. A similar effect concerning the formed multiscale conduction pathways responsible for the increase in conductivity of PA66/30CF/CNT composites, where CNTs provided a conductive pathway between discontinuous CFs, was described in [53]. In the case of the PVC matrix, the synergistic effect of the combination of two carbon nanostructures in a polymer composite containing single-walled carbon nanotubes (SWCNTs) and fullerene C60 increasing conductivity was described in [56].

### 2.7. Swelling Behaviour of Nanocomposites

Although PVC has good chemical resistance, it dissolves in ketones, ethers, and aromatic or chlorinated hydrocarbons [57] and undergoes limited swelling in acetone [24]. The characteristics of the swelling process may depend on the structure of the composites, i.e., the quality of dispersion of micro- and nanoparticles in the matrix and the strength of the interactions at the polymer–filler interface. Therefore, an analysis of the swelling process was used to assess the chemical resistance of PVC composites, and indirectly, it was also used to assess the structure and nature of polymer–filler interactions.

**Figure 8:**
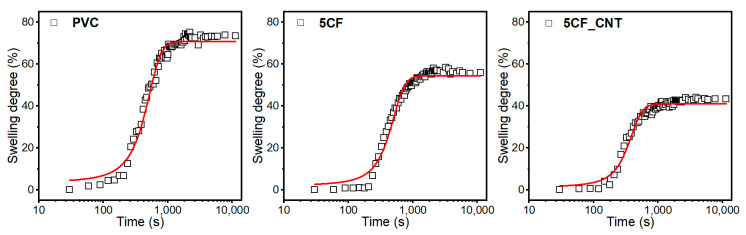
Swelling degree (S_d_) of PVC and PVC composites with 5% of CFs and 5% of CF_CNT as a function of time.

Figure 8 presents swelling curves as a function of time for PVC and selected composites, i.e., PVC/5CF and PVC/5CF_CNT.

It was found that all the materials obtained underwent limited swelling, while the dependence of the swelling degree on the exposure time to the swelling agent has the shape of a sigmoid function. Therefore, the function described by Equation (3) was used to approximate the swelling curves [24]. Table 4 summarises the parameters of Equation (3), and the coefficient of determination *R*^2^ indicates the degree of fit of the experimental results to the assumed model. The resulting high values of the coefficient of determination *R^2^* close to unity indicate that the proposed model describes with high accuracy the experimental results of the behaviour of PVC materials during swelling in acetone.

It was found that the CF- and CF_CNT-modified PVC composites were characterised by a lower degree of equilibrium swelling compared to PVC. Initially, up to a content of 5 wt.%, equilibrium swelling *(S_E_*) decreased with increasing filler content in the matrix, but at the maximum content, i.e., 10 wt.%, these values increased, regardless of whether CFs or the CF_CNT system was used. In addition, lower *S_E_* values were observed in composites where nanoparticles were used in the modification system. Compared to PVC, the PVC/5CF composite showed an increase in swelling resistance of approx. 24%, while the PVC/5CF_CNT composite showed an increase of 41.5%.

The lower *S_E_* value of composites with 5 wt.% CFs is related to its satisfactory homogeneous dispersion in the matrix without significant modifier aggregates. The homogeneous structure of the micromodifier in the matrix produced may physically hinder the access of solvent molecules to the macromolecules (blocking and impeding solvent penetration—barrier effects). In addition, the reduction in swelling can be linked to increased interfacial interactions between the microfiller and the polymer matrix, which has the effect of limiting the sorption capacity, i.e., reducing the amount of solvent that can diffuse into the matrix. Increasing the concentration of CF microfiller can lead to an increase in the amount of structure imperfections in the form of agglomerates or insufficient adhesion at the filler–matrix interface, resulting in increased *S_E_* values. Reduced interfacial adhesion can also lead to structure discontinuities, which can additionally act as sorption channels/tunnels, increasing the amount of diffusing solvent.

When a filler is used in a hybrid system of CFs and CNTs, the *S_E_* values are lower compared to composites modified with CFs only. The use of CNTs results in an increase in chemical resistance to swelling agents. This effect is related to the favourable dispersion of nanoparticles in the PVC matrix and their increased interaction with polymer macromolecules. Homogeneously dispersed nanoparticles cause an increase in interfacial interactions between the nanofiller and macromolecules, contributing to an increase in the rigidity of the structure and reducing intermolecular free spaces, thus increasing its packing density. This has the effect of reducing solvent availability to the macromolecules of the polymer chain [58].

## 3. Materials and Methods

### 3.1. Materials

As a polymer matrix, the composition of an unplasticized dry blend of poly(vinyl chloride) (PVC) was used. The components of the dry blend were as follows: PVC type S61 (Anwil SA, Włocławek, Poland) (100 phr), thermal stabiliser Mark MOK 17M (Acros, Renningen, Germany) (4 phr), and wax Loxiol G22 (Henkel, Dusseldorf, Germany) (1 phr).

Commercial milled carbon fibres (CFs) type PX 35, (ZOLTEK™ Toray Group, Bridgeton, MO, USA) with an average diameter of 7.2 μm and an average fibre length of 150 μm were used as a micromodifier. Industrial-grade multiwalled carbon nanotubes (MWCNTs) type NC7000 (Nanocyl S.A., Sambreville, Belgium) (average diameter of about 9.5 nm, an average length of 1.5 microns, and a maximum length of 50 μm) were used implemented as a carbon nanomodifier. Acetone with a purity of 99.5% (Pol-Aura, Dywity, Poland) was used as the sonication environment for a mixture of CFs and CNTs.

### 3.2. Preparation of CF_CNT Filler

To increase the dispersibility of CNTs in the PVC matrix with carbon fibre systems, a dispersion of carbon nanotubes in acetone was produced by sonication using a SONOPULS 3200HD homogeniser from Bandelin, equipped with a rod probe (VS 70 T). Dispersion was carried out for 30 min at a temperature of 20 °C by ultrasound with a frequency of 20 kHz and an amplitude of 40%, and the pulsation of the ultrasound was turn-on time: 0.4 s and turn-off time: 0.6 s. The resulting dispersion was then mixed with the CFs in acetone and mixed by a mechanical mixer (Ika Eurostar 6000) for 10 min. The resulting mixture was then dried for 24 h at 60 °C in a forced-air dryer and a further 24 h in a vacuum dryer at 60 °C until the residual acetone was completely removed. In this way, a micro-nanocarbon filler CF_CNT was obtained with a 20:1 component ratio in line with earlier work [30].

### 3.3. Preparation of PVC/CF-CNT Nanocomposites

Unmodified PVC and PVC composites were obtained by extrusion followed by compression moulding. For this purpose, a dry blend of PVC with process additives was produced (mixing time 15 min, mixing speed 50 min^−1^, temperature 100 °C). The dry blend obtained was then mixed with the filler using a mechanical mixer (Ika Eurostar 6000) (mixing time: 10 min). The resulting mixtures were extruded (Brabender laboratory extruder with a screw diameter of d = 15 mm and a length of L/D = 14). The extrusion temperature was as follows: 165 °C in zone I, 180 °C in zone II, and 185 °C at the head. The screw’s rotation speed was equal to 50 min^−1^, and a nozzle with a diameter of 2 mm and a length of 30 mm was used. The resulting pellets were compressed (temperature: 190 °C and maximum pressure: 15 MPa) to obtain plates with dimensions of 100 mm × 100 mm × 4 mm and 120 mm × 120 mm × 2 mm. The proportion of the filler system CF_CNT was 1, 5, and 10 wt.%. The same procedure was used to compare the effect of carbon nanoparticles on material properties, and PVC/CF composites were produced with the same concentration as above.

### 3.4. Material Characterisation

The structure of the fillers and the PVC composites was assessed using a scanning electron microscope Tescan MIRA3 (Tescan, Brno, Czech Republic), applying an accelerating voltage of 12 kV. In the case of PVC composites, the cryogenic breakthroughs of the samples were coated with a carbon coating of approximately 20 nm thickness.

The mechanical properties in static tension (elastic modulus, maximum stress, and strain at maximum stress) were determined according to EN ISO 527 (ISO 527-1:2012; Plastics-Determination of Tensile Properties-Part 1: General Principles. International Organization for Standardization: Brussels, Belgium, 2012) using a Zwick/Roell Z010 testing machine. Standardised specimens (type 5A) were used for the tests [59]. The test speed was 10 mm/min (for a modulus of 1 mm/min).

To investigate the thermal stability of the obtained materials, thermogravimetric tests (TGA) were carried out using a TG 209 F3 Netzsch Group (Germany, Selb) device in the temperature range of 30–900 °C at a rate of 10 °C·min^−1^ in an inert atmosphere. The temperature at which the 5% weight loss of the material (*T*_5_) was determined as the temperature of thermal stability. The temperature at which a 10% and 50% weight loss of the sample occurred was also determined (*T*_10_ and *T*_50_). The undecomposed residue of the material at 900 °C (*RM*) was also estimated, and the temperature of the maximum transformation rate (*T_DTG_*) was determined from the derivative thermogravimetric (DTG) curves.

By using the Congo red method, the time of thermal stability, indicating the initiation of PVC macromolecule decomposition and the evaporation of hydrogen chloride as a PVC decomposition product, was determined. A material was placed in a glass test tube, with Congo test paper inserted in the upper part of the test tube, and placed in an oil bath heated up to 200 °C. As a result of the test, the time after the first visible colour change of the indicator paper occurred was recorded.

The glass transition temperature of materials was estimated by the Differential Scanning Calorimetry (DSC) method using the DSC 204F1 Netzsch device (Selb, Germany). The material sample with an approximate weight of 25 mg was placed in the device’s chamber in a punctured crucible, and the measurement was then taken at the temperature range of 20–140 °C in a nitrogen atmosphere with two heating and cooling cycles. The analysis of the inflection point of the observed baseline change (*T_g Infl_*) and the half height of the incremental baseline change (*T_g Mid_*) from the second heating cycle were used to designate the glass transition temperature.

The Dynamic Mechanical Analysis (DMA) was performed to assess changes in the glass transition temperature, which was determined as designated based on the changes in the storage modulus (*E*′) as the start of a sudden drop in the value of *E*′ and on the loss angle tangent (tan *δ*) as a maximum value, as a function of temperature. The tests were conducted with the use of the DMA Artemis Netzsch device (Selb, Germany) in the mode of a three-point bending layout (support spacing of 20 mm, sample width of 10 mm, thickness of 1 mm), with a strain of 10 μm, a temperature range of 25–120 °C, a build-up speed of 2 °C·min^−1^, and a frequency equal to 1 Hz.

Based on the DMA results, the filler’s effect incorporated in the PVC matrix on the changes of *E’* modulus was calculated as a “C” coefficient [39,59] based on Equation (1):

(1) $$C=\frac{E_{g}^{\prime \mathrm{comp}}/E_{v}^{\prime \mathrm{comp}}}{E_{g}^{\prime \mathrm{PVC}}/E_{v}^{\prime \mathrm{PVC}}}$$

where *E*′_g_ and *E*’_v_ are the storage modulus measured in glassy (g) and viscoelastic (v) states for PVC (*E*′^PVC^) and PVC composites (*E*′^comp^). The modulus values were measured at 30 °C corresponding to the glassy state of materials and at 90 °C corresponding to the material’s viscoelastic state. Also are designated the changes in *E′* modulus at 25 °C, 40 °C, 60 °C, 80 °C, and 90 °C.

The electrical properties of composites were determined by testing the surface and volume resistivity. The measurement was accomplished by using a measurement system consisting of a 6517A electrometer and an 8009 measurement chamber (Keithley Instrument Inc., Cleveland, OH, USA) at 23 °C with a relative humidity of 50% and a voltage of 10 V.

The swelling resistance was tested in acetone following the method proposed in our earlier study [24]. The samples’ diameter changes (initially 10 mm) immersed in acetone at 20 °C were determined by application of the NIS Elements 4.0 software, which took pictures at a frequency of 1 every 10 s. On this basis, the changes in the swelling degree (*S_d_*), following Equation (2), were determined:

(2) $$S_{d}=\frac{h\;-\;h_{0}}{h_{0}}\;\times 100\%$$

where *h* is the sample diameter after time t (mm), and *h*_0_ is the initial sample diameter (mm).

The function described by Equation (3) [24] was used to approximate the dependence of the degree of swelling on the time of exposure to the swelling agent for the materials obtained that undergo limited swelling:

(3) $$S_{d}(t)=\frac{S_{E}}{1+10^{(t_{M}-t)p}}$$

where

*S_d_*—swelling degree, %;

*S_E_*—equilibrium swelling, upper asymptote, %;

*t_M_*—time in which the swelling occurs with a maximum rate, s;

*t*—time of exposure to the swelling agent, s;

*p*—comparison parameter, s^−1^.

## 4. Conclusions

The use of carbon nanotubes as an additional nanomodifier with a small percentage of CNTs in the polymer matrix can significantly alter the properties of a PVC–carbon fibre composite as a result of the formation of an additional spatial structure at the nanometric level (secondary nanosized network).

PVC composites manufactured with the simultaneous use of CFs and CNTs are characterised by a multiscale structure formed by micro- and nanofillers dispersed in the polymer matrix. PVC/CF_CNT composites show a higher stiffness and a higher *T_g_* value compared to PVC/CF. The lower mechanical strength is due to the adhesion at the interface being insufficient transfer higher stresses from the matrix to the fibres, but also to the presence of carbon aggregates that act as discontinuities in the composite structure. Thermal stability was slightly improved in multiscale composites.

Despite the structural defects revealed by SEM studies, the PVC/CF_CNT multiscale composites show increased resistance to swelling agents and improved electrical conductivity. Furthermore, the quality of the structure allowed for an increase in electron conductivity and gave the material new properties. The use of CNTs as an additional nanomodifier at a low proportion in the polymer matrix can significantly alter the properties of the composite, which is the result of the formation of an additional spatial structure at the nanometric level.

The CF_CNT filler preparation method allowed an increase in the dispersion quality of the nanofiller without the need for multistep composite preparation procedures.

As shown in the study, the use of CNTs as an additional modifier at the nanometric scale in PVC/CF composites can significantly improve some of the material properties, but further work is needed to maximise the achieved performance values while also aiming at improving the mechanical properties.

## Figures and Tables

**Table 1:** The T_g_ results of investigated materials.

Sample Name	*T_g_* Infl (°C)	*T_g_* Mid (°C)	*T_g_* Onset E′ (°C)	*T_g_* Max tan *δ* (°C)
PVC	75.7	75.9	75.2	92.3
1CF	75.4	75.8	76.3	93.3
5CF	75.8	75.8	77.1	94.3
10CF	75.8	75.9	78.2	94.8
1CF_CNT	75.7	75.9	76.8	94.4
5CF_CNT	75.6	75.5	77.6	94.9
10CF_CNT	76.2	76.4	79.3	95.5

**Table 2:** Storage modulus at different temperatures and values of coefficient C.

Sample Name	*E′* 30 °C (MPa)	*E′* 50 °C (MPa)	*E′* 70 °C (MPa)	*E′* 90 °C (MPa)	Coefficient C
PVC	3067	2892	2569	150	-
1CF	3634	3424	2778	215	0.83
5CF	4127	4016	3453	359	0.56
10CF	4364	4118	3707	467	0.46
1CF_CNT	3665	3433	2864	236	0.76
5CF_CNT	4225	4101	3561	388	0.53
10CF_CNT	4910	4562	4087	562	0.43

**Table 3:** The results of thermogravimetry and time of thermal stability by Congo red test.

Sample Name	*T*_1_	*T*_5_	*T*_10_	*T*_50_	*T*_DTG_	RM at 900 °C	Congo Test
	(°C)	(°C)	(°C)	(°C)	(°C)	(%)	(min)
PVC	236.7	254.5	270.4	313.8	291.5	11.30	32.8
1CF	236.5	257.9	274.2	316.2	292.4	13.00	32.3
5CF	237.5	260.1	276.4	318.2	291.1	16.80	33.4
10CF	239.2	262.4	278.1	321.5	291.0	21.60	34.3
1CF_CNT	234.9	258.0	274.7	315.4	291.2	12.60	33.8
5CF_CNT	236.9	259.1	275.0	319.6	292.2	16.70	34.2
10CF_CNT	237.3	259.7	275.6	321.7	292.4	21.30	34.9

**Table 4:** Model parameters describing the swelling process.

Sample	S_E_, (%)	t_M_, (s)	p, (s^−1^)	R^2^
PVC	70.7 (0.5)	432.6 (7.6)	0.003 (0.0001)	0.98
1 CF	63.2 (0.5)	446.7 (8.3)	0.003 (0.0001)	0.98
5 CF	54.3 (0.4)	442.5 (8.9)	0.003 (0.0002)	0.98
10 CF	61.8 (0.6)	432.5 (10.9)	0.002 (0.0001)	0.97
1CF_CNT	60.9 (0.4)	460.3 (8.8)	0.002 (0.0001)	0.98
5CF_CNT	40.9 (0.3)	335.1 (7.5)	0.004 (0.0003)	0.97
10CF_CNT	52.7 (0.4)	496.9 (8.6)	0.002 (0.00008)	0.99

(…)—standard deviations are given in brackets.

## Data Availability

The data that support the findings of this study are available from the corresponding author on request.
